# MRI findings in human rabies: A case report on the importance of neuroimaging when biological tests are inconclusive

**DOI:** 10.1016/j.radcr.2025.03.056

**Published:** 2025-04-12

**Authors:** Zakaria Chahbi, Said Adnor, Soufiane Bigi, Mounir Salek, Soukaina Wakrim

**Affiliations:** Department of Radiology, University Hospital of Agadir, Faculty of Medicine and Pharmacy of Agadir, IBN ZOHR University, Agadir, Morocco

**Keywords:** meningitis, Viral, Encephalitis, Rabies, MRI

## Abstract

Rabies is a rare but fatal viral infection affecting the central nervous system, often presenting with nonspecific symptoms that can delay diagnosis. We report the case of a 14-year-old boy who presented with fever, headache, neck stiffness, photophobia, and convulsions, initially mimicking other central nervous system infections. Brain and spine MRI revealed hyperintense signals in the bilateral basal ganglia, thalami, limbic cortex and brainstem on T2-weighted and FLAIR images, without restricted diffusion on diffusion-weighted imaging and an MR spectroscopy consistent with rabies encephalitis. Spinal cord MRI showed an hyperintense signal that resolved on follow-up imaging after one month. This case highlights the role of MRI in detecting early neuroimaging changes in rabies encephalitis and underscores the need for clinical correlation for timely diagnosis and management. Further studies are needed to better define the utility of MRI in rabies encephalitis.

## Introduction

Rabies is a fatal viral infection that affects the central nervous system, and it is transmitted to humans through the bite of infected animals. Once the virus reaches the brain, it causes inflammation and damage to the neural tissues, leading to encephalitis and ultimately resulting in a fatal outcome if left untreated [1]. The diagnosis of rabies is usually based on clinical suspicion, and laboratory confirmation of the virus which is often difficult due to the short window of opportunity for treatment. Therefore, imaging plays a crucial role in the diagnosis and management of rabies encephalitis [2].

Although imaging findings of rabies on MRI are not specific, they can help in detecting early changes in the brain and guiding appropriate management. In this case report, we present the imaging findings of a child with rabies encephalitis who underwent MRI of the brain and spine. The purpose of this report is to describe the imaging features of rabies on MRI, which can aid in the diagnosis and management of this rare and deadly infection.

## Patient and observation

A 14-year-old male with no significant past medical or surgical history presented to the hospital with fever, headache, and seizures. He had no known chronic illnesses, history of immunosuppression, or recent travel. His family history was unremarkable for neurological or autoimmune disorders. Two weeks prior to the onset of symptoms, the patient was bitten by a stray dog on the lower extremity. He promptly received postexposure prophylaxis, including the full course of rabies vaccination according to WHO guidelines, but did not receive rabies immunoglobulin.

The illness began with low-grade fever, generalized fatigue, and intermittent headaches, which progressively worsened over the course of a week. He subsequently developed photophobia, neck stiffness, and confusion, followed by multiple generalized tonic-clonic seizures, prompting hospital admission. On examination, he was febrile (38.5°C), tachycardic (110 bpm), and had moderate hypertension (140/90 mmHg). Neurological examination revealed nuchal rigidity, bilateral papilledema, cranial nerve deficits (including facial weakness and dysphagia. There were no focal motor deficits or signs of autonomic dysfunction at admission.

Initial laboratory investigations showed the following:

Complete blood count (CBC):

White blood cell count: 12, 500/mm³ (normal: 4, 000–11, 000/mm³)
Hemoglobin: 13.2 g/dL (normal: 12–16 g/dL)
Platelets: 280, 000/mm³ (normal: 150, 000–450, 000/mm³)

Inflammatory markers:

C-reactive protein (CRP): 8 mg/L (normal: <5 mg/L)
Erythrocyte sedimentation rate (ESR): 22 mm/hr (normal: <20 mm/hr)

Cerebrospinal fluid (CSF) analysis:

Appearance: Clear
White blood cells: 2 cells/mm³ (normal: 0–5 cells/mm³)
Protein: 42 mg/dL (normal: 15–45 mg/dL)
Glucose: 60 mg/dL (normal: 50–80 mg/dL)
Viral PCR panel, including herpes simplex virus and enteroviruses: Negative
Rabies PCR on CSF: Negative

**Fig. 1:**
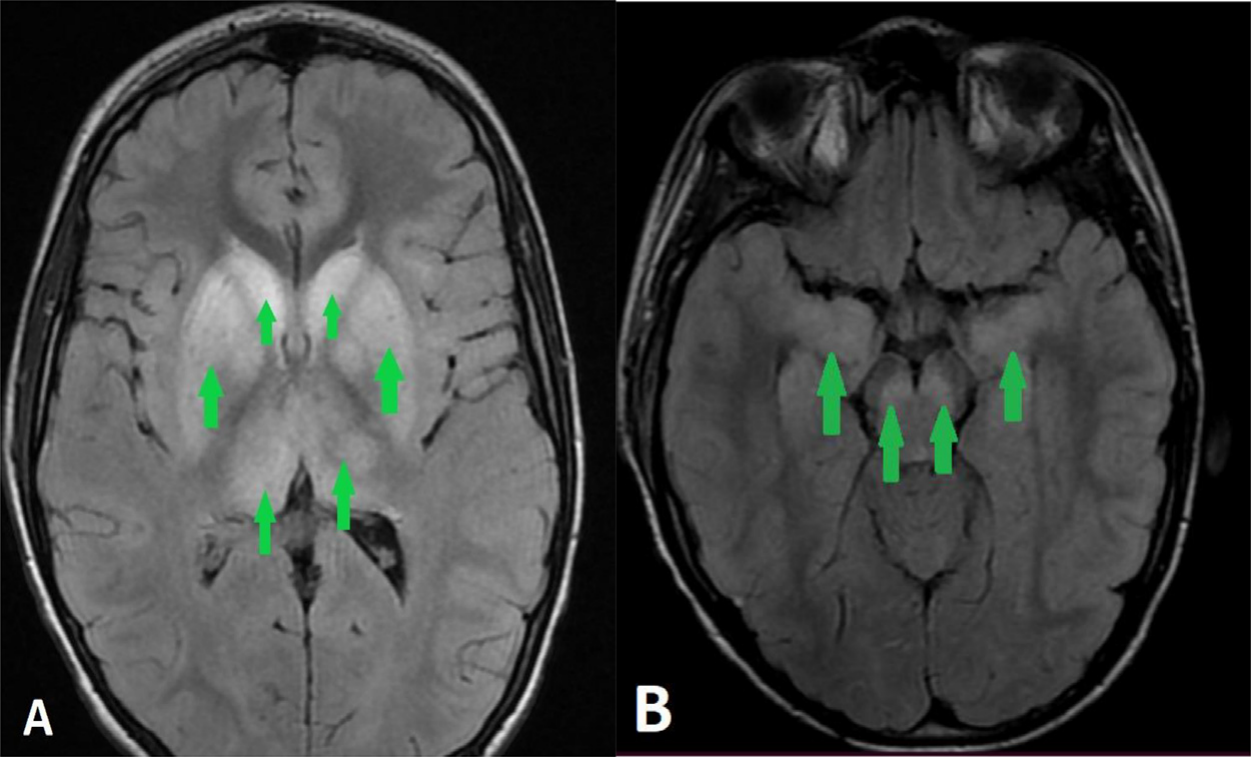
T2 FLAIR sequence showing a symmetrical hypersignal of basal ganglia (A), limbic cortex and substantia nigra (B) (green arrows).

**Fig. 2:**
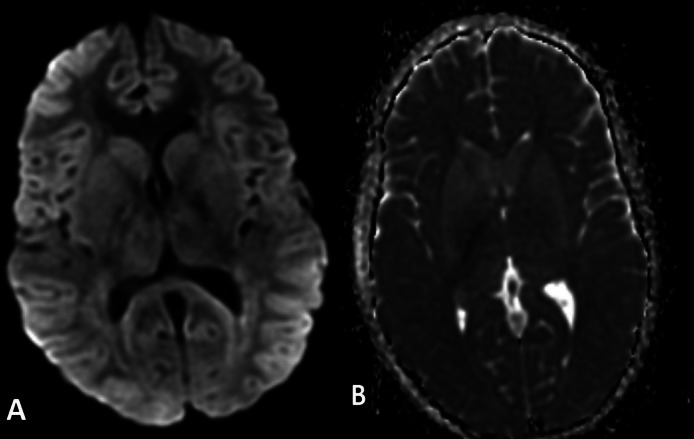
DWI sequence (A) with ADC (B) showing no restricted diffusion.

**Fig. 3:**
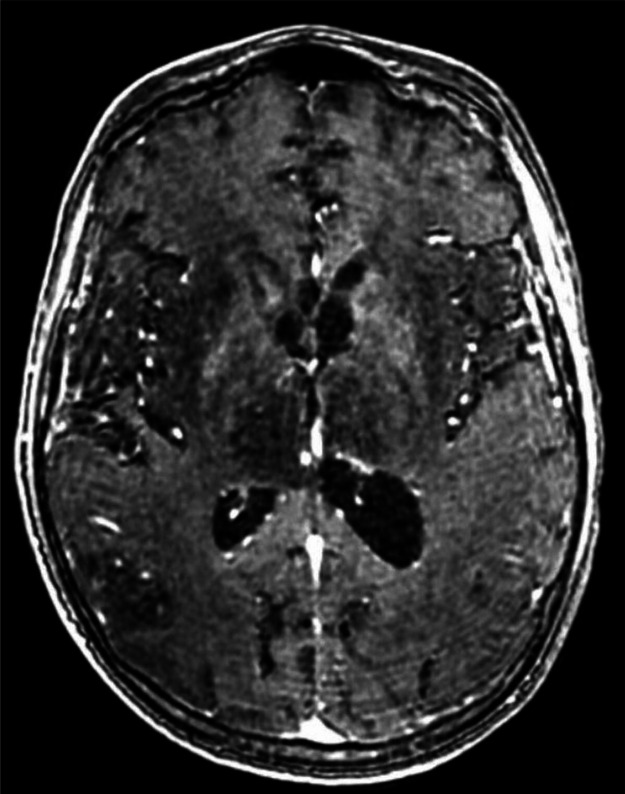
T1 sequence with Gadolinium injection sowing no enhancement of the basal ganglia.

**Fig. 4:**
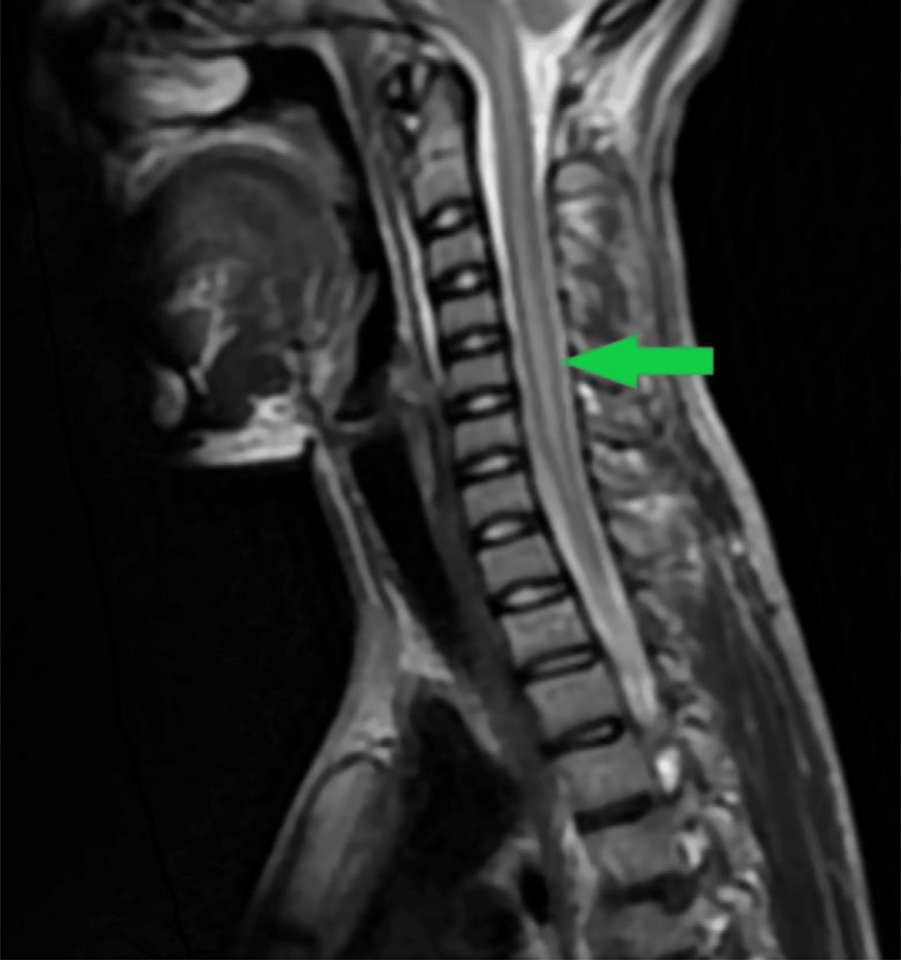
T2 medullar sequence showing an hypersignal of the cervical spinal cord (green arrow).

An initial noncontrast CT scan of the brain was unremarkable. However, MRI revealed diffuse FLAIR hyperintensity in the basal ganglia, substantia nigra, and limbic cortex (Fig. 1) without restricted diffusion (Fig. 2) or gadolinium enhancement (Fig. 3), alongside a T2 hypersignal in the spinal cord (Fig. 4). These findings suggested viral encephalitis or acute disseminated encephalomyelitis (ADEM) secondary to vaccination. Given the concern for ADEM, MOG antibodies were tested, but results were negative, reducing the likelihood of an autoimmune etiology. The patient was started on intravenous acyclovir for suspected viral encephalitis and broad-spectrum antibiotics (ceftriaxone and vancomycin) while awaiting further diagnostic results.

Despite aggressive supportive and antiviral treatment, the patient's condition deteriorated. He developed agitation, trismus, hypersalivation, and episodes of autonomic instability, including fluctuating blood pressure and tachyarrhythmia.

**Fig. 5:**
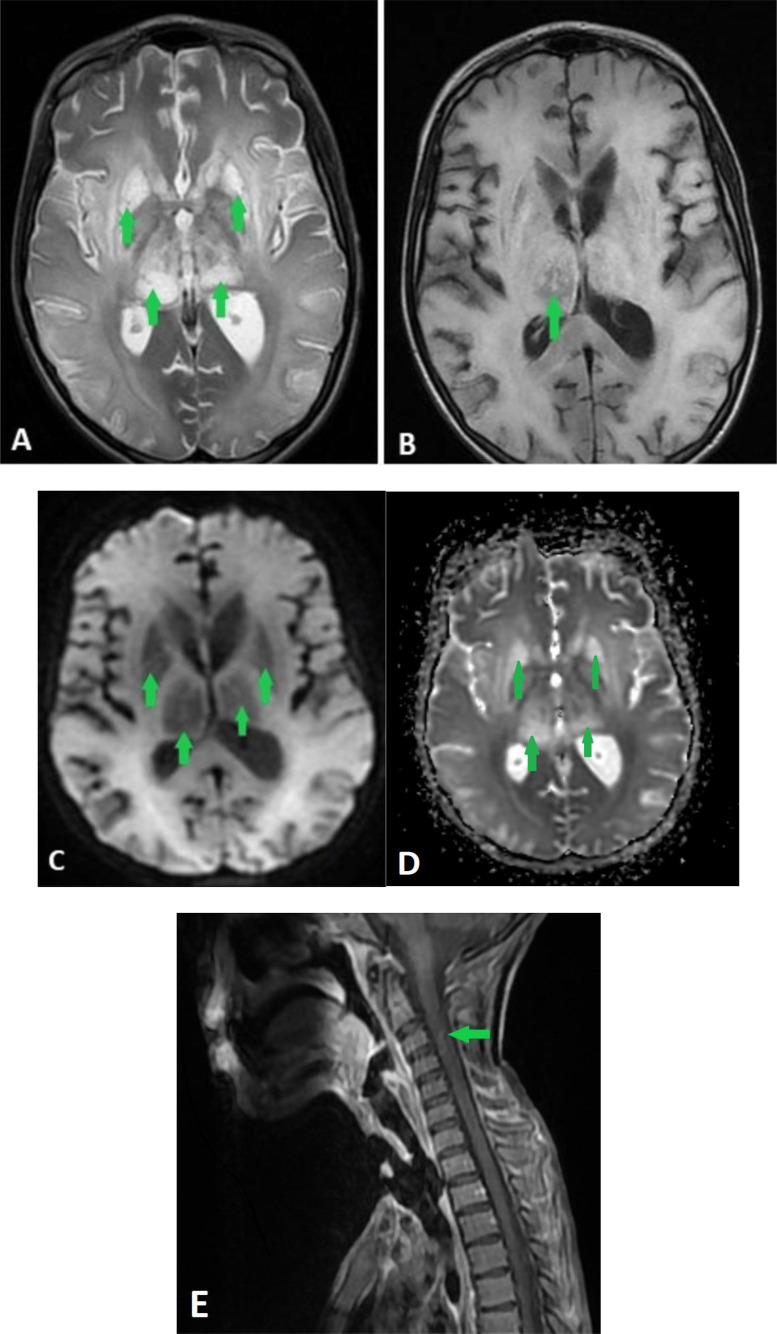
Control MRI after 1 month showing the vasogenic edema in the basal ganglia due to the late stage of the disease. A: T2 hypersignal of basal ganglia (green arrow), B: FLAIR hyposignal of right thalamus (green arrow), C: Diffusion hyposignal of basal ganglia (green arrow), D: facilitated ADC in basal ganglia E: T1 hyposignal of cervical spinal cord (green arrow).

A repeat MRI conducted one month later (Fig. 5) revealed the progression of the diffuse FLAIR hyperintensity in the basal ganglia, substantia nigra, and limbic cortex without gadolinium enhancement, alongside a T2 hypersignal in the spinal cord. Diffusion-weighted imaging showed facilitated diffusion due to extensive vasogenic edema.

**Fig. 6:**
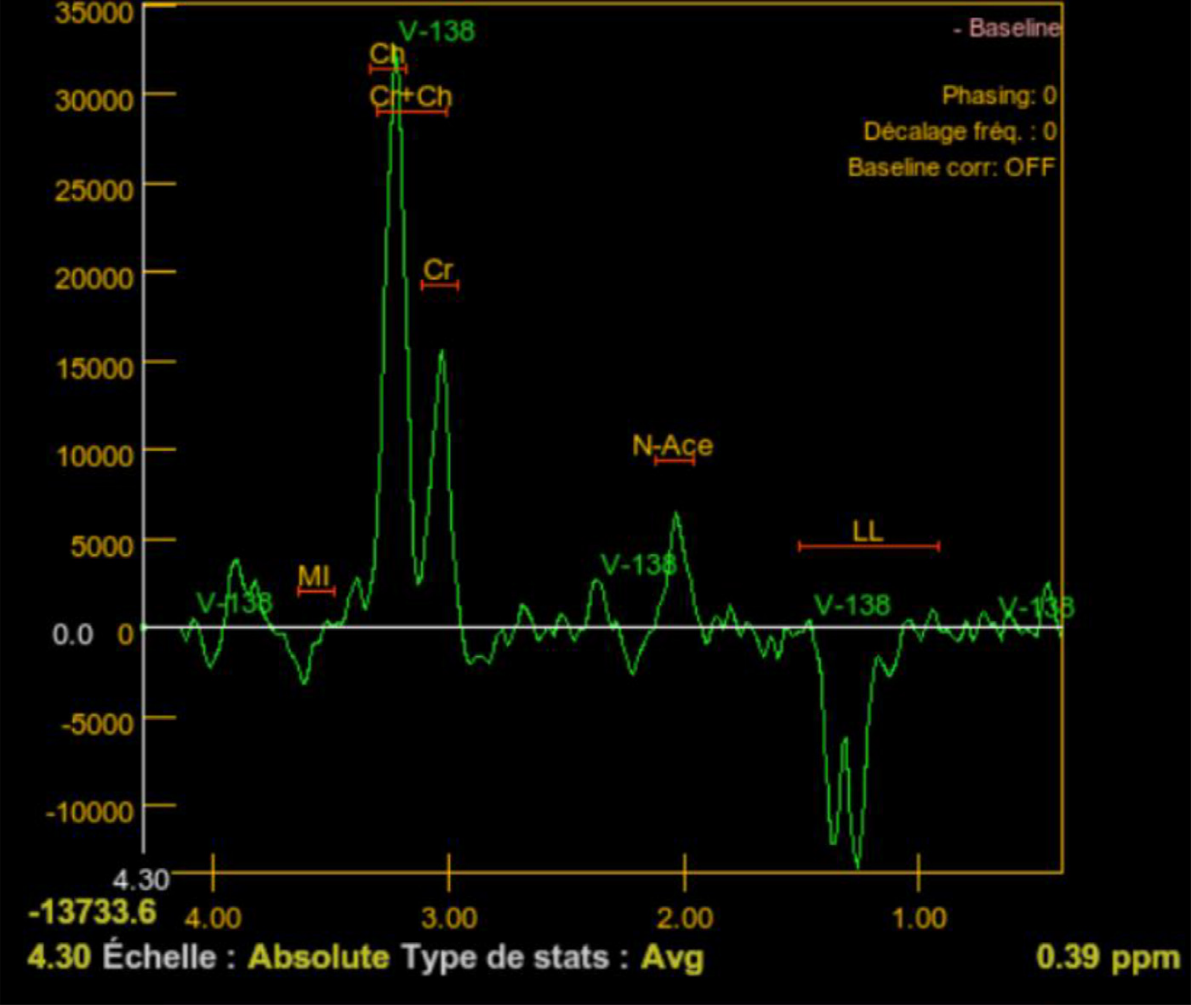
MR Spectroscopy with long TE of 144 ms done 1 month after the onset of symptoms showing marked reduction in N-acetyl aspartate (NAA), elevated choline (Cho) peak, myo-inositol elevated level (mI) and a prominent lipid and lactate (LL) peak.

Magnetic resonance spectroscopy (MRS) performed during the disease course (Fig. 6) on basal ganglia demonstrated a marked reduction in N-acetyl aspartate (NAA), indicative of neuronal loss, along with an elevated choline (Cho) peak, reflecting increased membrane turnover due to inflammation and gliosis. Additionally, myo-inositol (mI) levels were elevated, suggesting an active glial response. A prominent lipid and lactate (LL) peak was observed, with an inverted lactate signal at long TE (144 ms), consistent with anaerobic metabolism and tissue hypoxia.

The patient's respiratory function progressively worsened, requiring mechanical ventilation. Unfortunately, he succumbed to respiratory failure within weeks of symptom onset. This case underscores the diagnostic challenges of rabies encephalitis, particularly in vaccinated individuals with negative PCR results. It highlights the importance of considering rabies in atypical presentations and the potential role of advanced imaging in early diagnosis

## Discussion

Rabies encephalitis is a rare but deadly infection that primarily affects the central nervous system. The virus is transmitted through the saliva of infected animals, and once it enters the body, it travels through the peripheral nerves to reach the brain, where it causes severe inflammation and damage to the neural tissues. The clinical presentation of rabies encephalitis is often nonspecific and can mimic other infectious and noninfectious neurological diseases, making the diagnosis challenging [1]. In such cases, neuroimaging plays a crucial role in establishing the diagnosis and guiding appropriate management.

The imaging findings in rabies encephalitis are not pathognomonic, and they may overlap with other viral encephalitis. However, the involvement of specific regions of the brain and spinal cord, along with the clinical history and other laboratory findings, can aid in the diagnosis of rabies encephalitis. Magnetic resonance imaging (MRI) is the preferred imaging modality for the evaluation of rabies encephalitis, as it provides excellent soft tissue contrast and spatial resolution.

The MRI findings in our case report showed hyperintense signals in the bilateral basal ganglia, thalami, and brainstem on T2-weighted and fluid-attenuated inversion recovery (FLAIR) images, along with facilitated diffusion in the same areas on diffusion-weighted imaging (DWI). These findings are consistent with the previous reports of rabies encephalitis and are thought to be due to the rabies virus's tropism for the neurons and glial cells of these regions [[2], [3]]. The DWI findings suggest cytotoxic edema, indicating that the rabies virus causes damage to the neurons, leading to their swelling and eventual death, however, in the late stages of the disease, we tend to have facilitated diffusion which is due to vasogenic oedema and neural loss similarly to our case. Additionally, the absence of spinal cord involvement on MRI is not uncommon in cases of rabies encephalitis [4].

The involvement of the basal ganglia, thalami, and brainstem on MRI is a characteristic feature of rabies encephalitis, and it distinguishes it from other viral encephalities, such as herpes simplex encephalitis and Japanese encephalitis [5]. The involvement of the brainstem in rabies encephalitis is particularly important, as it can lead to respiratory failure and death [6]. Moreover, the MRI findings in rabies encephalitis often precede the clinical symptoms, which emphasizes the importance of early diagnosis and treatment in improving the patient's outcome [6].

Other imaging modalities, such as computed tomography (CT) and positron emission tomography (PET), have also been used in the evaluation of rabies encephalitis. CT may reveal nonspecific findings such as cerebral edema and ventricular enlargement, while PET may show hypometabolism in the affected regions. However, these modalities are less sensitive than MRI and may not provide the same level of detail in the evaluation of rabies encephalitis.

Advanced MRI techniques, such as diffusion-weighted imaging (DWI) and diffusion tensor imaging (DTI), offer greater sensitivity than conventional MRI in detecting abnormalities at the molecular level. These methods can reveal both micro- and macrostructural damage and generate measurable maps of mean diffusivity (MD) and fractional anisotropy (FA) in the brain. When compared to normal brain maps, these quantitative assessments can highlight statistically significant abnormalities, including swelling and restricted interstitial spaces.

Additionally, MR spectroscopy can detect biochemical changes associated with neuronal damage, glial cell injury, and cell membrane breakdown. These techniques can also be used for disease monitoring and progression analysis by comparing MD and FA values and MR spectral ratios over time [2].

The imaging findings on MRI, such as hyperintense signals in the basal ganglia, thalami, and brainstem, along with restricted diffusion in the beginning then facilitated diffusion, are characteristic of rabies encephalitis and aid in the diagnosis of the disease. The involvement of the brainstem on MRI is particularly important in predicting the outcome of the disease. Early diagnosis and appropriate management are crucial in improving the patient's outcome, and neuroimaging plays a crucial role in achieving this goal [6].

## Conclusion

In conclusion, this case report of human rabies infection detected through MRI imaging serves as a poignant reminder of the importance of timely and accurate diagnosis in the management of neurological diseases. The clinical presentation of rabies can be nonspecific and often mimics other encephalitic infections, making early recognition and treatment challenging. The use of advanced imaging techniques such as MRI can aid in the diagnosis and management of complex neurological conditions, including viral infections such as rabies.

This case highlights the need for increased public education and vaccination programs to control the spread of this deadly disease. Rabies remains a significant public health concern in many parts of the world, particularly in regions where canine vaccination rates are low. Therefore, it is crucial that healthcare professionals maintain a high index of suspicion for rabies in patients presenting with neurological symptoms, and advocate for comprehensive rabies vaccination and control measures in their communities. Ultimately, improved awareness and effective control strategies will help prevent further needless deaths from this preventable and treatable disease.

## Patient consent

The patient agreed to have the article written and published.
